# Aneurysmal Bone Cyst of the Radial Diaphysis Managed by En Bloc Resection and Fibular Strut Autograft Reconstruction in a Young Adult Female: A Rare Case Report

**DOI:** 10.1002/ccr3.73828

**Published:** 2026-10-30

**Authors:** Abdul Hadi Khan, Emman Hussain, Laiba Arif, Syed Owais Akhtar, Ahmed Asad Raza, Abedin Samadi, Nadeem Ahmed

**Affiliations:** ^1^ Jinnah Postgraduate Medical Center Karachi Pakistan; ^2^ Department of Orthopaedic Surgery Jinnah Postgraduate Medical Center Karachi Pakistan; ^3^ Department of Medicine Jinnah Sindh Medical University Karachi Pakistan; ^4^ Department of Medicine Kabul University of Medical Sciences Abu Ali Sina Kabul Afghanistan

**Keywords:** aneurysmal bone cyst, case report, en bloc resection, fibular strut graft, radial diaphysis

## Abstract

Aneurysmal bone cyst (ABC) of the radial diaphysis is rare and may mimic telangiectatic osteosarcoma on imaging. We report a young woman with progressively enlarging, painful forearm swelling due to ABC, treated by en bloc resection and fibular strut autograft reconstruction, achieving pain‐free union, good function and no recurrence at short‐term follow‐up.

## Key Clinical Message

Aneurysmal bone cyst should be considered when evaluating an expansile lytic lesion of the radial diaphysis in a young adult, despite imaging overlap with telangiectatic osteosarcoma. Histopathological correlation is essential before treatment. En bloc resection with fibular strut autograft reconstruction is a reasonable option for large, aggressive diaphyseal lesions; in this patient it achieved local control, graft union and good forearm function, with a modest loss of rotation at short‐term follow‐up, although longer surveillance is required.

**Abbreviations**
ABC: aneurysmal bone cyst
CE‐MRI: contrast‐enhanced magnetic resonance imaging
CT: computed tomography
HPF: high‐power field
LCP: locking compression plate
MRI: magnetic resonance imaging
MSTS: Musculoskeletal Tumor Society
OPD: outpatient department
QuickDASH: shortened Disabilities of the Arm, Shoulder and Hand questionnaire

## 1 Introduction

Aneurysmal bone cyst (ABC) is a benign but locally aggressive osteolytic bone lesion characterized by blood‐filled spaces separated by fibrous septa containing fibroblasts, osteoclast‐type giant cells, and reactive woven bone [1]. Although ABCs are uncommon, accounting for approximately 1% of primary bone tumors, and most cases occur during the first two decades of life [1, 2].

ABC most commonly involves the metaphyseal regions of long bones and the posterior elements of the vertebral column [1, 2]. Clinically, patients usually present with localized pain, swelling, or tenderness, and a pathological fracture [2]. Plain radiographs typically demonstrate an eccentric, expansile, lytic lesion with cortical thinning and a well‐defined margin. Magnetic resonance imaging (MRI) may demonstrate fluid–fluid levels, which may also be observed in other lesions, including telangiectatic osteosarcoma [3]. Therefore, correlation between clinical, radiological, and histopathological findings is essential for accurate diagnosis.

Treatment options for ABC include intralesional curettage with or without bone grafting or local adjuvants, en bloc resection, and image‐guided sclerotherapy [4]. Although the prognosis is generally favorable, local recurrence remains an important concern, particularly in younger patients and in lesions managed with curettage alone [1, 4].

ABC may affect any part of a long bone, but involvement of the radial shaft is uncommon [5]. Lesions in this location may present diagnostic and therapeutic challenges because of the functional importance of the radius in forearm rotation and upper‐limb function. We present a rare case of an aneurysmal bone cyst of the radial shaft in a young adult female, highlighting its clinical presentation, radiological and histopathological evaluation, management, and clinical outcome.

## 2 Case Presentation

### 2.1 Patient Information

A 23‐year‐old, right‐hand‐dominant woman presented to the orthopedic outpatient department with swelling of the left forearm of 9 months' duration and pain in the same region for the preceding 4 months. The swelling had been noticed incidentally and had enlarged gradually to approximately half the size of a tennis ball, without any antecedent trauma and without overlying skin changes. Over the last 4 months, the swelling had become painful. The pain was throbbing and intermittent in nature, nonradiating, aggravated by physical activity, and relieved by oral analgesics. There were no constitutional symptoms such as fever, night sweats, anorexia, or weight loss and no history of similar swellings elsewhere in the body.

Her past medical and surgical history was unremarkable, with no known comorbid illness and no long‐term medication. Family history, including any history of skeletal or neoplastic disease, was noncontributory and her psychosocial history was unremarkable. She had received no prior intervention for the swelling other than intermittent self‐administered analgesics, which provided only temporary relief.

### 2.2 Clinical Findings

On general physical examination, the patient was comfortable and afebrile, with no swelling in any other part of the body and an otherwise unremarkable systemic examination. On local musculoskeletal examination, there was a single oval swelling measuring approximately 8 × 3 cm over the anterior aspect of the left mid‐forearm (Figure 1A). The overlying skin was normal in color and temperature, with no redness, dilated veins, or ulceration, and was freely mobile over the swelling. The swelling was firm in consistency, well defined, nonmobile, and fixed to the underlying structures. Mild tenderness was elicited on deep palpation. No bruit was audible on auscultation over the swelling. Elbow, forearm, and wrist movements were preserved. The regional lymph nodes were not palpable, and the distal neurovascular status of the limb was intact.

**FIGURE 1:**
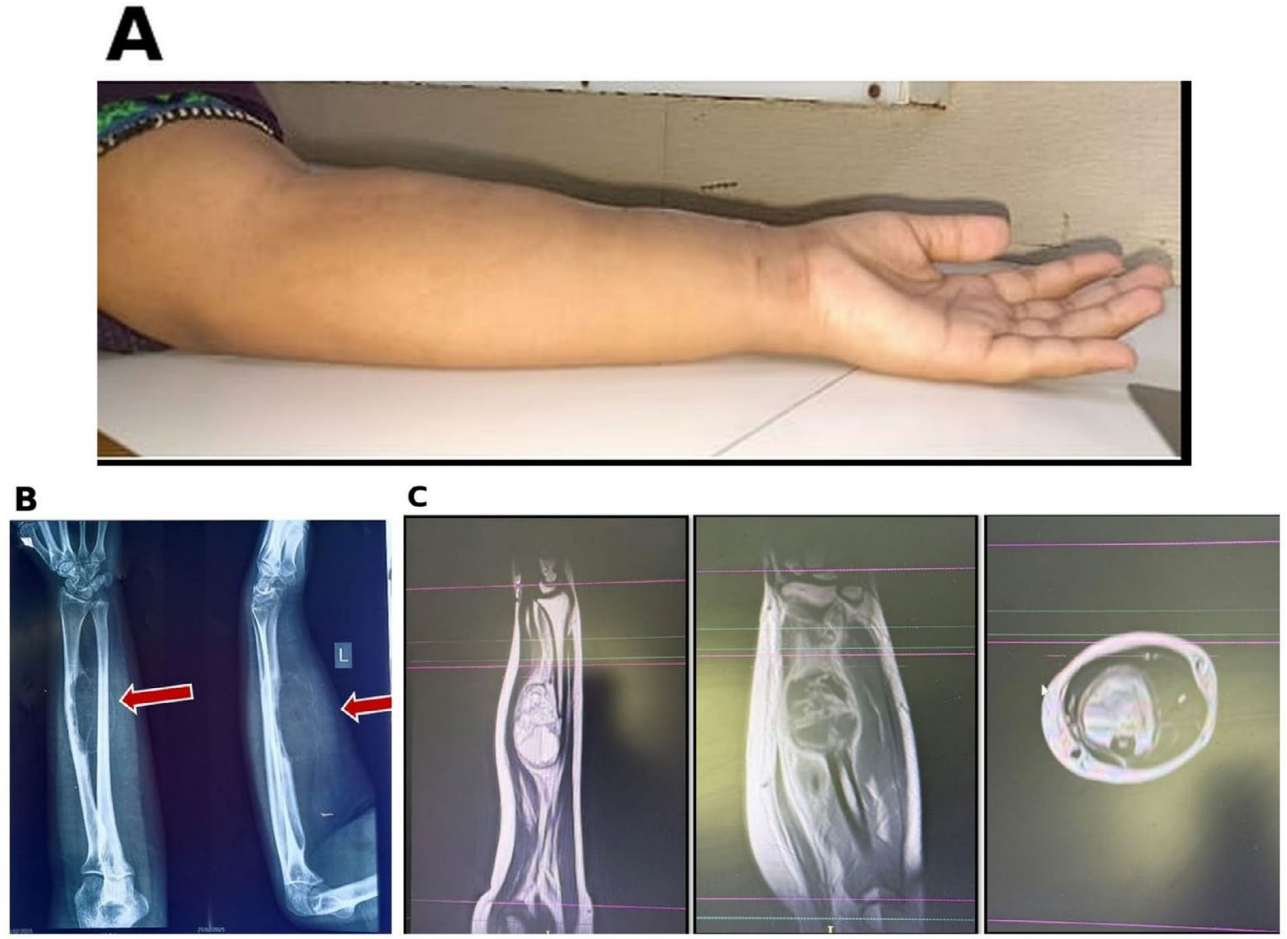
Preoperative clinical and radiological assessment of the left forearm. (A) Clinical photograph showing a swelling over the anterior aspect of the left mid‐forearm with normal overlying skin. (B) Anteroposterior and lateral radiographs of the left forearm demonstrating an eccentric, expansile radiolucent lesion of the mid radial shaft with septate loculations and cortical thinning (arrows). (C) Contrast‐enhanced MRI of the left forearm (sagittal, coronal, and axial images) showing a lobulated lesion of the volar compartment with multiple fluid–fluid levels, cortical erosion of the radial diaphysis, and displacement of the adjacent neurovascular structures.

### 2.3 Timeline

The chronological sequence of the episode of care is summarized in Table 1.

**TABLE 1:** Timeline of clinical presentation, diagnostic work‐up, intervention, and follow‐up.

Time point	Event and clinical findings
9 months before presentation	Painless swelling first noticed over the anterior aspect of the left mid‐forearm; no antecedent trauma.
4 months before presentation	Swelling progressively enlarged to about half a tennis‐ball size and became painful; throbbing, intermittent, nonradiating pain aggravated by activity and relieved by analgesics.
Presentation (day 0)	Orthopedic outpatient assessment; 8 × 3 cm firm, fixed swelling of the anterior mid‐forearm; distal neurovascular status intact; baseline laboratory investigations within normal limits.
Week 1	Plain radiographs and contrast‐enhanced MRI of the left forearm.
Week 2	Three‐phase skeletal scintigraphy; contrast‐enhanced CT of the chest and whole abdomen for staging.
Week 3	Incisional biopsy of the lesion; histopathology consistent with aneurysmal bone cyst in correlation with radiological findings.
Week 7 (day of surgery)	En bloc resection of the involved segment of the radial diaphysis through a modified Henry approach; reconstruction of the 11 cm defect with a nonvascularised fibular strut autograft and locking compression plate fixation.
Postoperative week 2	Wound healed primarily; sutures removed; above‐elbow immobilization continued.
Postoperative week 4	Final histopathology of the resected specimen reported as the solid variant of aneurysmal bone cyst with uninvolved margins.
Postoperative week 6	Immobilization discontinued after radiographic review; graded physiotherapy commenced.
Postoperative month 3	Pain‐free; radiographs showed progressive incorporation of the graft.
Postoperative month 6	Complete union; pain‐free; normal elbow and wrist motion with residual loss of forearm rotation (pronation 60°, supination 65°); MSTS score 27/30; QuickDASH score 18; no radiological evidence of recurrence.

### 2.4 Diagnostic Assessment

All baseline laboratory investigations, including complete blood count, erythrocyte sedimentation rate, serum calcium, phosphate, alkaline phosphatase, and renal and liver function tests, were within normal limits.

Plain radiographs of the left forearm in anteroposterior and lateral projections demonstrated an eccentric ballooning expansion of the mid radial shaft with radiolucency and septate loculations, associated with cortical thinning (Figure 1B). Contrast‐enhanced MRI of the left forearm showed a well‐defined lobulated lesion of abnormal signal intensity with multiple internal fluid–fluid levels in the anterior (volar) compartment of the mid forearm, measuring 3.5 × 3.7 × 6 cm (anteroposterior × transverse × craniocaudal), with associated cortical erosion involving the mid radial diaphysis (Figure 1C). The soft tissue component partially encircled the bone with mild extension into the posterior compartment and anteriorly displaced the forearm muscles. The lesion showed heterogeneous enhancement with a central nonenhancing area, thick fascial enhancement extending from the tumor margins, producing a tail sign, and peritumoral edema encasing the interosseous vessels with displacement of the median nerve and radial vessels. The reported radiological differential diagnoses were soft tissue sarcoma with telangiectatic change, telangiectatic osteosarcoma, and subperiosteal aneurysmal bone cyst.

Three‐phase skeletal scintigraphy performed with technetium‐99m methylene diphosphonate showed increased blood flow and blood pool accumulation of tracer over the left forearm, with a focal area of increased radiotracer uptake over the mid shaft of the left radius on delayed images; the remainder of the axial and appendicular skeleton showed symmetrical uptake. The findings indicated active bone pathology of the mid radial shaft suggestive of a primary bone tumor requiring histopathological correlation. Contrast‐enhanced CT of the chest and whole abdomen, performed as part of the staging work‐up, showed no evidence of pleuropulmonary metastasis; a few axillary lymph nodes with preserved fatty hila and small hypodense hepatic lesions with marginal vascularity, consistent with hemangiomas, were noted as incidental findings.

For definitive characterization, an incisional biopsy was performed. Microscopic examination revealed fragments of a neoplastic lesion comprising abundant osteoclast‐like multinucleated giant cells between which spindle‐shaped cells were interspersed. The individual cells had moderate cytoplasm and round to oval nuclei with variably prominent nucleoli, and mitotic activity of up to 3 per 10 high‐power fields was appreciated. Areas of hemorrhage with cystic change were also present. Immunohistochemistry for H3F3A G34W (H3G34W) was negative, and no malignant osteoid was identified. The features were reported as consistent with aneurysmal bone cyst in correlation with the radiological findings. Because a limited incisional sample cannot entirely exclude telangiectatic osteosarcoma or a secondary aneurysmal bone cyst arising in another lesion, the definitive diagnosis was reserved for examination of the complete resected specimen (Section 2.6). The diagnostic investigations and their key findings are summarized in Table 2.

**TABLE 2:** Summary of diagnostic investigations and key findings.

Investigation	Key findings	Interpretation
Laboratory work‐up	Complete blood count, erythrocyte sedimentation rate, serum calcium, phosphate, alkaline phosphatase, renal and liver function tests within normal limits.	No systemic or metabolic bone disease.
Plain radiographs (forearm, AP and lateral)	Eccentric ballooning expansion of the mid radial shaft with radiolucency, septate loculations, and cortical thinning.	Expansile lytic lesion of the radial diaphysis.
Contrast‐enhanced MRI	Lobulated lesion, 3.5 × 3.7 × 6 cm, in the volar compartment of the mid‐forearm; multiple fluid–fluid levels; cortical erosion of mid radial diaphysis; heterogeneous enhancement with central nonenhancing area; fascial tail sign; displacement of median nerve and radial vessels.	Neoplastic lesion with a hemorrhagic component; differentials: soft tissue sarcoma with telangiectatic change, telangiectatic osteosarcoma, and subperiosteal ABC.
Three‐phase skeletal scintigraphy	Increased blood flow and blood pool over the left forearm; focal increased uptake over the mid shaft of the left radius on delayed images; remainder of the skeleton unremarkable.	Solitary active bone lesion of the mid radial shaft; no multifocal skeletal involvement.
CT chest and whole abdomen	No pleuropulmonary metastasis; reactive axillary nodes with fatty hila; small hepatic hemangiomas.	No evidence of distant metastatic disease.
Incisional biopsy	Abundant osteoclast‐like multinucleated giant cells with interspersed spindle cells; up to 3/10 HPF mitoses; hemorrhage with cystic change; H3G34W immunostain negative.	Consistent with aneurysmal bone cyst; negative H3G34W argued against giant cell tumor of bone when interpreted with the histological and radiological features.
Histopathology of resected specimen	Solid variant of aneurysmal bone cyst measuring 7 × 3 × 1.5 cm; all soft tissue and bone margins uninvolved (bone margins 1.0 cm proximally and 0.8 cm distally).	Complete excision; no malignant transformation.

### 2.5 Diagnostic Challenges and Differential Diagnosis

The principal diagnostic challenge in this case was the nonspecific nature of the imaging findings. Multiple fluid–fluid levels, cortical breach, a soft tissue component, and peritumoral edema are all features shared by aneurysmal bone cyst and telangiectatic osteosarcoma, and the initial radiological report favored a malignant etiology. The differential diagnosis therefore included telangiectatic osteosarcoma, soft tissue sarcoma with secondary bone involvement, giant cell tumor of bone, unicameral bone cyst, and fibrous dysplasia. Sequential investigation was required, with imaging, skeletal scintigraphy, staging CT, and finally tissue diagnosis obtained at separate visits, which contributed to an interval of several weeks between presentation and definitive surgery. Giant cell tumor of bone was considered unlikely on the combined clinical, radiological, and histological picture rather than on immunohistochemistry alone: the lesion was centered in the diaphysis without epiphyseal or subarticular extension, it contained multiple fluid–fluid levels with hemorrhagic cystic change, and the osteoclast‐like giant cells were admixed with spindle cells rather than evenly distributed within sheets of mononuclear cells, as is characteristic of giant cell tumor. The negative H3G34W immunostain was supportive; however, because a small proportion of giant cell tumors harbor H3F3A mutations other than G34W and are negative with this antibody, the result was interpreted only in conjunction with the morphological and radiological findings and was not regarded as excluding giant cell tumor in isolation. Telangiectatic osteosarcoma was excluded on examination of the entire resected specimen, which showed no malignant osteoid, no marked nuclear pleomorphism, and no atypical mitotic figures, and the final diagnosis was a solid variant of aneurysmal bone cyst of the radial diaphysis.

### 2.6 Therapeutic Intervention

Intralesional curettage with bone grafting was initially considered, but was not undertaken because of the size of the lesion, the extent of cortical destruction of the diaphysis, the presence of a soft tissue component abutting the neurovascular bundle, and the radiological possibility of a telangiectatic osteosarcoma. Although the incisional biopsy favored aneurysmal bone cyst, several considerations led the treating team to prefer en bloc resection. First, the lesion was large, with cortical erosion of the mid‐radial diaphysis and an extraosseous soft tissue component that partially encircled the bone; curettage would have left a thin, structurally incompetent cortical shell at high risk of pathological fracture and would still have required structural grafting and internal fixation. Second, the soft tissue component was closely related to the median nerve and the radial and interosseous vessels, making thorough intralesional clearance and the safe use of local adjuvants (high‐speed burring, phenol, or cryotherapy) difficult without risk to these structures. Third, recurrence after intralesional treatment is more frequent in large, aggressive lesions, and recurrent disease adjacent to the neurovascular bundle would have made any subsequent resection more hazardous [4]. Fourth, the imaging had raised concern for telangiectatic osteosarcoma, and the biopsy represented only a limited sample; en bloc resection with a cuff of normal tissue therefore provided definitive treatment and complete histological assessment without compromising margins had a malignant diagnosis emerged. Finally, the diaphyseal location allowed resection without sacrificing the elbow or wrist articulations, leaving an intercalary defect amenable to biological reconstruction. En bloc resection was therefore selected as the definitive procedure in order to obtain wide margins and minimize the risk of local recurrence.

The procedure was performed under general anesthesia with the patient supine, the affected limb on an arm table, and an arm tourniquet applied. Perioperative intravenous antibiotic prophylaxis was administered at induction. The lesion was exposed through a modified Henry approach to the volar forearm. The lesion, which measured approximately 10 × 6 cm in situ, including its extraosseous soft tissue component and the surrounding reactive tissue, was identified (Figure 2A). The radial artery and the median nerve were identified and protected throughout the dissection, and the affected segment was carefully isolated and removed en bloc with a cuff of surrounding normal tissue (Figure 2B). The resected specimen was sent for histopathological examination.

**FIGURE 2:**
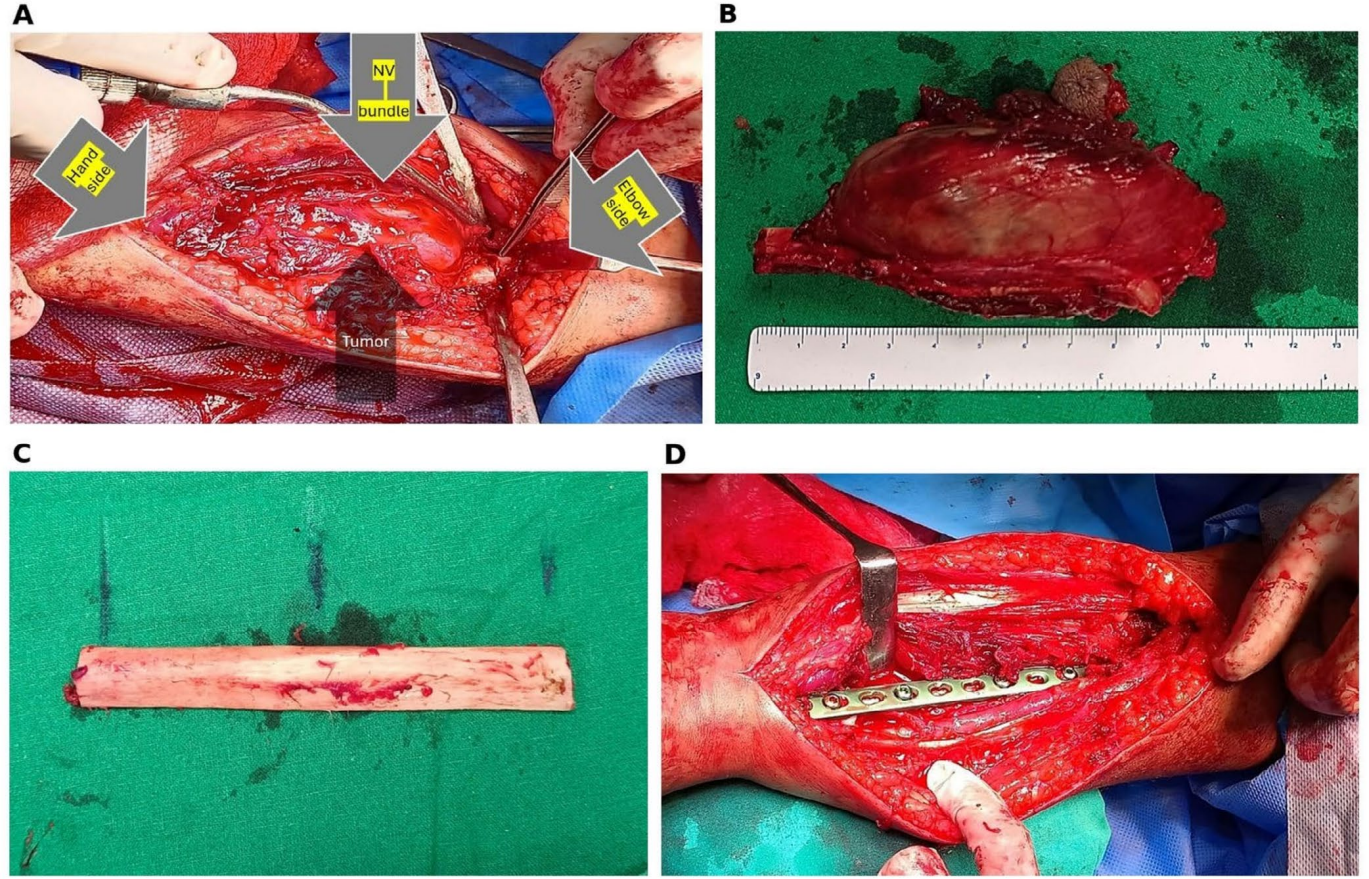
Intraoperative photographs. (A) The lesion exposed through a modified Henry approach, with the tumor arising from the radial diaphysis and the adjacent neurovascular bundle retracted and protected. (B) The en bloc resected specimen with the involved segment of the radial diaphysis and its soft tissue component, shown alongside a centimeter scale. (C) Nonvascularised fibular strut autograft harvested for reconstruction of the segmental defect. (D) The strut graft seated within the radial defect and stabilized with a locking compression plate.

Following resection, a segmental bone defect of approximately 11 cm remained (see below for the relationship between lesion size and defect length). Reconstruction was performed using a nonvascularized fibular strut autograft (Figure 2C), which was contoured to the defect, seated within the proximal and distal radial stumps, and stabilized with a locking compression plate (Figure 2D). Intraoperative imaging confirmed satisfactory alignment and fixation. The tourniquet was released, hemostasis secured, and the wound closed in layers over a suction drain. The limb was immobilized in an above‐elbow slab.

Histopathological examination of the entire resected specimen demonstrated a lesion measuring 7 × 3 × 1.5 cm composed predominantly of solid cellular areas of bland spindle‐shaped fibroblastic cells with scattered osteoclast‐like multinucleated giant cells, reactive woven bone, and foci of hemorrhage, with only a minor component of blood‐filled cystic spaces separated by fibrous septa. The predominance of these solid areas over the classic cystic architecture formed the basis for the designation of a solid variant of aneurysmal bone cyst. There was no marked nuclear pleomorphism, no atypical mitotic figures, and no malignant osteoid, and no background lesion suggesting a secondary aneurysmal bone cyst was identified. All soft tissue and bone margins were uninvolved.

The different dimensions reported for the lesion reflect different methods and stages of measurement. The clinical dimension (8 × 3 cm) was a surface estimate of the palpable swelling, including the overlying soft tissue. The MRI dimensions (3.5 × 3.7 × 6 cm) describe the well‐defined lesion itself, with approximately 6 cm of craniocaudal extent along the radius. The intraoperative estimate (approximately 10 × 6 cm) was made in situ before resection and included the extraosseous soft tissue component, the reactive pseudocapsule, and surrounding edematous tissue. The pathological dimension (7 × 3 × 1.5 cm) was measured on the formalin‐fixed specimen, after drainage of the blood‐filled spaces and fixation‐related shrinkage. The length of the bone defect (approximately 11 cm) was determined by the resection levels rather than by lesion size alone: the osteotomies were placed beyond the longitudinal extent of abnormal signal on MRI to obtain a margin of normal bone, because telangiectatic osteosarcoma had not been excluded preoperatively, and the radial ends were then freshened to healthy, bleeding cortical bone to permit stable graft–host contact. The pathological bone margins (1.0 cm proximally and 0.8 cm distally) were measured on the fixed specimen and are therefore expected to be smaller than the corresponding in vivo distances.

### 2.7 Follow‐Up and Outcomes

The postoperative course was uncomplicated. The wound healed primarily and the sutures were removed at 2 weeks. The limb was immobilized for 6 weeks, after which radiographs were obtained and a graded physiotherapy program comprising active and active‐assisted elbow, forearm, and wrist mobilization followed by progressive strengthening was commenced. The patient adhered fully to the immobilization protocol and the physiotherapy program and attended all scheduled follow‐up visits at 2 weeks, 6 weeks, 3 months, and 6 months. No adverse or unanticipated events occurred; in particular, there was no surgical site infection, no postoperative neurovascular deficit, no implant failure, and no donor site morbidity.

Serial radiographs demonstrated progressive incorporation of the strut graft. At 6 months of follow‐up, the patient reported no pain and had returned to her routine daily activities. Goniometric range of motion of the operated left upper limb was 145° of elbow flexion and 0° of extension, and 77° of wrist flexion and 70° of wrist extension, all within normal limits, and 60° of forearm pronation and 65° of supination, giving a rotation arc of 125° with a residual deficit in both pronation and supination. The Musculoskeletal Tumor Society (MSTS) upper‐extremity score was 27/30 (90%) [6] and the QuickDASH score was 18 [7], indicating a good functional outcome with mild residual disability. Radiographs at that visit demonstrated complete union at both graft–host junctions with maintained alignment and intact fixation, and no radiological evidence of local recurrence (Figure 3).

**FIGURE 3:**
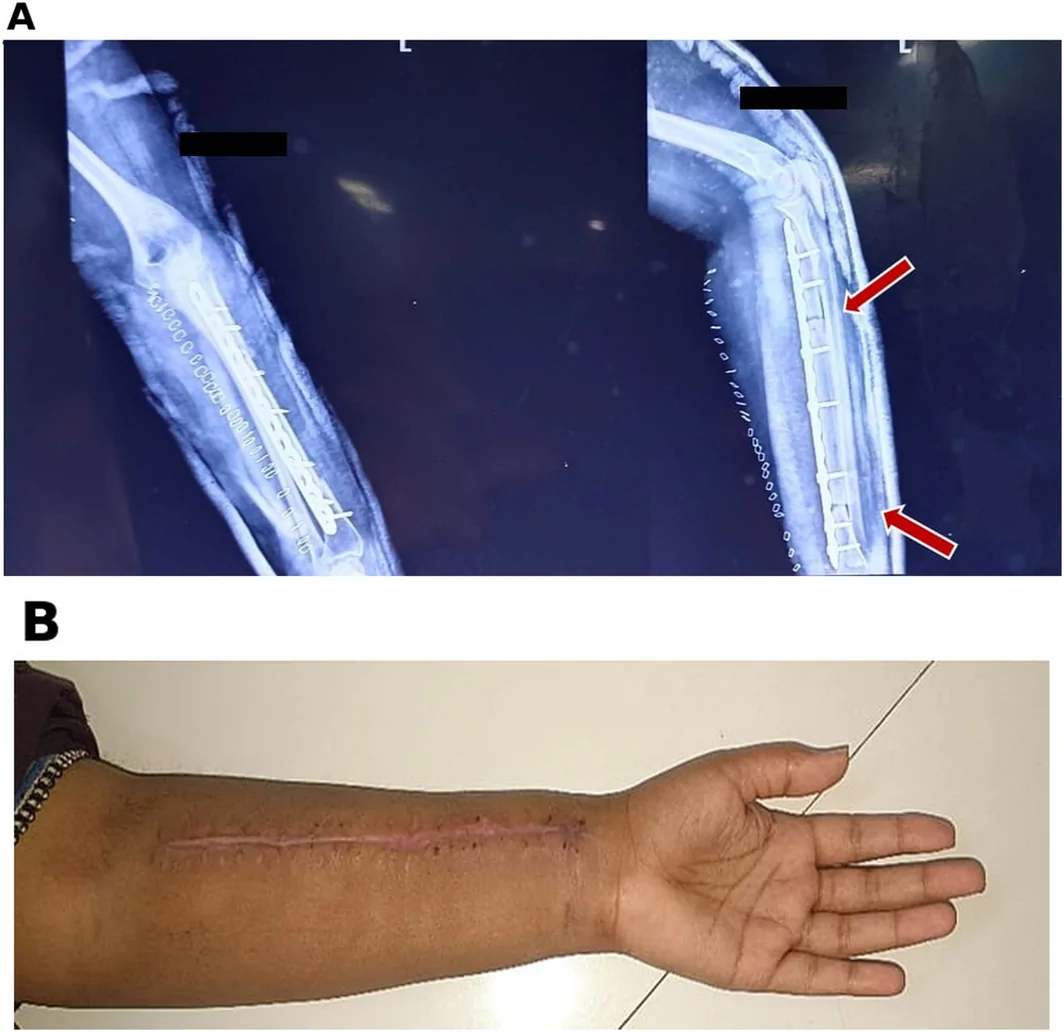
Postoperative assessment at 6 months. (A) Anteroposterior and lateral radiographs of the left forearm showing the fibular strut autograft bridging the segmental defect of the radius, stabilized with a locking compression plate, with union at the graft–host junctions (arrows), maintained alignment, and no evidence of recurrence. (B) Clinical photograph of the left forearm showing a well‐healed volar surgical scar and preserved forearm and hand contour.

### 2.8 Patient Perspective

The patient reported that she had initially ignored the swelling because it was painless, and that she sought medical advice only when the pain began to interfere with her daily activities. She described considerable anxiety during the diagnostic period, when the possibility of a malignant tumor was raised and several investigations were required before a diagnosis could be reached. She found the explanation of the surgical plan and of the reasons for removing a segment of bone reassuring, and stated that being told the final biopsy showed a benign lesion was the most important moment in her treatment. She reported that the period of immobilization was inconvenient but tolerable, and that she was satisfied with the outcome, as she is now free of pain, able to use her left arm normally for household and occupational tasks, and comfortable with the appearance of the scar.

## 3 Discussion

### 3.1 Clinical and Radiological Considerations

Aneurysmal bone cyst is an uncommon benign bone lesion that predominantly affects children and young adults. Although benign histologically, the lesion can behave aggressively and cause substantial bone destruction, cortical expansion, pathological fracture, and functional impairment. The present case is notable because of the unusual involvement of the radial shaft. ABCs more frequently involve the metaphyseal regions of long bones, whereas diaphyseal involvement is less common. Because of its unusual location, the lesion may mimic other expansile lytic lesions of bone. Radiological evaluation plays an important role in the assessment of ABC. Plain radiographs typically demonstrate an expansile, eccentric, lucent lesion with cortical thinning and a well‐defined margin. MRI provides additional information regarding the extent of the lesion and may demonstrate characteristic fluid–fluid levels caused by blood products of different ages within the lesion [8].

However, fluid–fluid levels are not specific for ABC and can also be seen in other lesions, including telangiectatic osteosarcoma, giant cell tumor, and other expansile lytic lesions of bone. Therefore, histopathological confirmation is important, particularly when imaging findings are atypical or the lesion demonstrates aggressive features.

The solid variant of ABC, in which solid fibroblastic and giant cell‐rich areas predominate over blood‐filled cystic spaces, adds further diagnostic difficulty because, on limited biopsy material, it may resemble giant cell tumor, giant cell reparative granuloma, or osteosarcoma. Its diagnosis therefore relies on assessment of the entire lesion and, where available, demonstration of a USP6 gene rearrangement, which is shared by conventional and solid ABC [8]. Similarly, H3F3A G34W immunohistochemistry is a useful adjunct in distinguishing giant cell tumor from giant cell‐rich mimics, but a negative result should be interpreted together with the patient's age, lesion location, imaging, and morphology rather than as an isolated exclusion criterion.

### 3.2 Management Considerations

Several treatment options have been described for ABC, including curettage, extended curettage with local adjuvant therapy, bone grafting, en bloc resection, embolization, and other minimally invasive techniques. The choice of treatment depends on factors such as lesion location, size, cortical integrity, risk of fracture, and proximity to important neurovascular structures [9, 10].

In the present case, complete (en bloc) surgical excision of ABC is used. This technique is associated with excellent local control rate and has been associated with lower recurrence rates than intralesional procedures in several reported series; however, its use may be limited by the potential for greater surgical morbidity and functional impairment [4, 11].

In our patient, en bloc resection was chosen despite a biopsy favoring ABC because of the combination of lesion size, cortical destruction, an extraosseous component abutting the neurovascular bundle, the anticipated recurrence risk after curettage, and residual preoperative concern for telangiectatic osteosarcoma. A closely comparable approach was reported by Regmi et al., who treated a 10‐year‐old boy with a recurrent aggressive ABC of the proximal radius by en bloc resection, vascularised fibular graft reconstruction, and anconeus interposition arthroplasty, with good clinical and radiographic results at 6 months [12]. Our case differs in that the lesion was primary rather than recurrent, the patient was skeletally mature, and the lesion was confined to the diaphysis, so that both the elbow and wrist articulations could be preserved without joint reconstruction. These factors allowed an intercalary reconstruction with a nonvascularised fibular strut autograft, which avoids microvascular anastomosis and shortens operating time. However, nonvascularised grafts, however, incorporate by creeping substitution and are generally considered to carry a higher risk of delayed union, stress fracture, and graft resorption than vascularised grafts, particularly for longer defects; vascularised fibular grafts are therefore often preferred for long defects, recurrent lesions, or a compromised soft tissue bed. Although union was achieved at both graft–host junctions by 6 months in our patient, an 11‐cm non‐vascularised graft remains at risk of late fatigue fracture during remodeling. Functionally, our patient regained normal elbow and wrist motion but had a residual loss of forearm rotation (arc of 125°), which may reflect the length of the resected segment and the difficulty of reproducing the radial bow with a straight fibular strut; nevertheless, her MSTS and QuickDASH scores indicated a good functional outcome.

### 3.3 Limitations

The main limitation of this report is that it represents a single case and therefore cannot establish the superiority of one treatment modality over another. Nevertheless, the unusual anatomical location provides useful clinical information for clinicians evaluating expansile osteolytic lesions of the radius.

In addition, the follow‐up period of 6 months is short. Although it was sufficient to document graft–host union and early functional recovery, most recurrences of ABC occur within the first 2 years after treatment, and incorporation and remodeling of a long nonvascularised fibular graft continue well beyond this period [4]. The absence of recurrence and the graft outcome reported here should therefore be regarded as short‐term results, and the patient remains under clinical and radiological surveillance. Finally, molecular confirmation by USP6 rearrangement analysis was not performed, and the diagnosis of the solid variant rests on morphological, immunohistochemical, and radiological correlation.

## 4 Conclusion

Aneurysmal bone cyst of the radial shaft is rare and can resemble other aggressive bone lesions. Accurate diagnosis requires correlation of clinical, radiological, and histopathological findings. In selected large, aggressive diaphyseal lesions, en bloc resection with nonvascularised fibular strut autograft reconstruction achieved graft union, a good functional outcome with a modest loss of forearm rotation, and no recurrence at short‐term follow‐up; longer follow‐up is required to confirm durable local control and long‐term graft incorporation.

## Author Contributions

**Abdul Hadi Khan:** conceptualization, writing – original draft, writing – review and editing, visualization. **Emman Hussain:** writing – original draft, writing – review and editing, supervision. **Laiba Arif:** writing – original draft, writing – review and editing. **Syed Owais Akhtar:** writing – original draft, writing – review and editing. **Ahmed Asad Raza:** supervision, writing – original draft, writing – review and editing, investigation. **Abedin Samadi:** writing – original draft, writing – review and editing. **Nadeem Ahmed:** conceptualization, supervision, validation, visualization, writing – original draft, writing – review and editing, project administration.

## Funding

The authors have nothing to report.

## Ethics Statement

Institutional ethical approval was not required for the publication of this single case report in accordance with institutional policy. The report was prepared in accordance with the ethical standards of the institution and with the principles of the Declaration of Helsinki.

## Consent

Written informed consent was obtained from the patient for the publication of this case report and the accompanying clinical images. All identifying information has been removed and a copy of the consent form is available for review by the Editor‐in‐Chief of this journal on request.

## Conflicts of Interest

The authors declare no conflicts of interest.

## Data Availability

Data sharing not applicable to this article as no datasets were generated or analyzed during the current study.
